# Aspalathin and Other Rooibos Flavonoids Trapped α-Dicarbonyls and Inhibited Formation of Advanced Glycation End Products In Vitro

**DOI:** 10.3390/ijms232314738

**Published:** 2022-11-25

**Authors:** Katarzyna Bednarska, Izabela Fecka

**Affiliations:** 1 Department of Pharmacognosy and Herbal Medicines, Faculty of Pharmacy, Wroclaw Medical University, ul. Borowska 211, 50-556 Wroclaw, Poland; 2 The Committee on Therapeutics and Pharmaceutical Sciences, The Polish Academy of Sciences, pl. Defilad 1, 00-901 Warszawa, Poland

**Keywords:** methylglyoxal, glyoxal, advanced glycation end products, trapping of dicarbonyls, *Aspalathus linearis*, rooibos, dihydrochalcones, flavonoids, MGO, AGEs

## Abstract

The excessive dietary intake of simple sugars and abnormal metabolism in certain diseases contribute to the increased production of α-dicarbonyls (α-DCs), such as methylglyoxal (MGO) and glyoxal (GO), the main precursors of the formation of advanced glycation end products (AGEs). AGEs play a vital role, for example, in the development of cardiovascular diseases and diabetes. *Aspalathus linearis* (Burman f.) R. Dahlgren (known as rooibos tea) exhibits a wide range of activities beneficial for cardio-metabolic health. Thus, the present study aims to investigate unfermented and fermented rooibos extracts and their constituents for the ability to trap MGO and GO. The individual compounds identified in extracts were tested for the capability to inhibit AGEs (with MGO or GO as a glycation agent). Ultra-high-performance liquid chromatography coupled with an electrospray ionization mass spectrometer (UHPLC–ESI–MS) was used to investigate α-DCs’ trapping capacities. To evaluate the antiglycation activity, fluorescence measurement was used. The extract from the unfermented rooibos showed a higher ability to capture MGO/GO and inhibit AGE formation than did the extract from fermented rooibos, and this effect was attributed to a higher content of dihydrochalcones. The compounds detected in the extracts, such as aspalathin, nothofagin, vitexin, isovitexin, and eriodictyol, as well as structurally related phloretin and phloroglucinol (formed by the biotransformation of certain flavonoids), trapped MGO, and some also trapped GO. AGE formation was inhibited the most by isovitexin. However, it was the high content of aspalathin and its higher efficiency than that of metformin that determined the antiglycation and trapping properties of green rooibos. Therefore, *A. linearis*, in addition to other health benefits, could potentially be used as an α-DC trapping agent and AGE inhibitor.

## 1. Introduction

Nowadays, it is well established that diet can significantly affect the metabolic health of the human body. In recent years, dietary habits have evolved all over the world, and the consumption of simple carbohydrates in the diet of the average person in developed countries has increased dramatically [1]. The increase in the prevalence of cardio-metabolic diseases, including obesity, type 2 diabetes, and the vascular complications of diabetes, is thought to be associated with a significant spike in the consumption of simple sugars, especially glucose and fructose. One of the mechanisms linking the increased intake of monosaccharides with the development of metabolic disorders is the overproduction of α-dicarbonyl compounds (α-DCs), such as methylglyoxal (MGO) and glyoxal (GO) [2,3]. Both MGO and GO are formed by the non-enzymatic and enzymatic degradation of monosaccharides; therefore, they may be produced in excess during the sustained consumption of large amounts of carbohydrates [4]. An elevated glucose metabolism may result in increased MGO formation mainly through the polyol pathway [5]. Moreover, lipid peroxidation with the increased production of highly reactive aldehydes, including glyoxal, may occur as a result of oxidative stress and lipid disorders induced by hyperglycemia [6]. However, glucose is not the only contributor to α-DC formation. The hepatic metabolism of fructose skips the regulated steps of glycolysis, which additionally impairs normal lipid and carbohydrate metabolism and results in the increased production and generation of MGO [7]. The concentration of methylglyoxal is therefore significantly higher in people with diabetes and on average is up to 2–4 times higher [8]. α-DCs are characterized by extremely high reactivity and the ability to interact with primary amine and guanidine groups of biomacromolecules by nucleophilic addition to form Schiff bases [9]. Subsequently, Schiff bases can undergo cyclization to produce glycosylamines, which are further rearranged to produce stable ketoamines (Amadori products) [10]. Both Schiff bases and Amadori products are products of reversible reactions. However, they can also be further transformed by oxidation, dehydration, polymerization, and oxidative degradation reactions, giving origin to advanced glycation end products (AGEs) [11]. The non-enzymatic protein glycation reaction described above can be triggered not only by α-DCs but also by reducing sugars (e.g., glucose and fructose), which contain electrophilic carbonyl groups [12].

However, it is assumed that α-DC activity exceeds that of the reduction of sugars by up to several hundred times. The process of non-enzymatic glycation occurs under physiological conditions in the body [13]. Nevertheless, since α-DCs are essentially derived through the metabolism of carbohydrates, the higher the glycemia, the more the non-enzymatic glycation process is enhanced [14]. This is particularly important in patients with poorly controlled, long-term hyperglycemia, as increased concentrations of both α-DCs and AGEs contribute significantly to the development and progression of the vascular complications of diabetes and other metabolic disorders [14]. Albumins are especially vulnerable to glycation modifications induced by high concentrations of simple sugars and α-DCs [15]. In a population of healthy individuals, the percentage of glycated albumin is estimated to range from 1 to 10%, whereas in a population of individuals with the vascular complications of diabetes, it may be as high as 30% [16]. Among the many mechanisms of interference in the glycation pathway proposed as being able to reduce the concentration of α-DCs and thus, in the long run, AGEs, one of the most promising besides lowering carbohydrate intake seems to be trapping α-DC compounds [17]. In the past, there were great hopes for the clinical utility of aminoguanidine, the first known methylglyoxal scavenger and AGE inhibitor. The ability of aminoguanidine to delay the progression of the vascular complications of diabetes, including retinopathy and nephropathy, has been demonstrated, as has its ability to inhibit protein glycation modifications in vivo. Nevertheless, further clinical trials were discontinued due to some serious side effects occurring after long-term treatment, including gastrointestinal disorders and anemia [18].

In continuing the search for anti-α-DC treatments, plants and isolated natural compounds have received increasing attention in recent years [19,20,21]. *Aspalathus linearis* (Burman f.) R. Dahlgren, also known as rooibos or redbush tea, is a popular plant raw material widely used around the world, and its beneficial health properties, especially in the context of cardiometabolic health, are fairly well recognized [22]. This plant is cultivated for its herbal tea, which occurs in two varieties, unfermented (green rooibos, GR) and fermented (red rooibos, RR) [23]. The hypoglycemic properties of *A. linearis* have been previously demonstrated; therefore, it is particularly recommended for patients with pre-diabetic and diabetic conditions as a complementary treatment [24]. However, the positive effects of *A. linearis* on the cardiovascular system are not limited to potential glucose-lowering activity. A study by Marnewick et al. [25] revealed that the regular consumption of traditional rooibos tea significantly improved lipid profile as well as redox status, which are both important for vascular health. In fact, the benefits associated with the use of rooibos tea appear to be far more extensive and according to new studies may also include the reduction of oxidative stress [26], inflammation [27], insulin resistance [28], protein glycation [29], and the alleviation of pancreatic β-cell dysfunction [30]. Nevertheless, despite such intensive research on the cardio-metabolic benefits of *A. linearis* extracts, to date, it has not been evaluated whether their mechanism of action also includes methylglyoxal and glyoxal trapping. Of particular interest in this context seems to be aspalathin, the main and predominant component in green rooibos, for which α-DC-trapping activity has not yet been demonstrated. However, the ability to uptake MGO has been reported in structurally similar compounds, including phloretin and phlorizin (apple constituents), which may suggest that aspalathin and *A. linearis* may possess analogous activity [31].

With this in mind, the aim of the present study is to investigate the MGO- and GO-trapping capacity of aspalathin and other phytochemicals present in the infusions and hydroethanolic extracts of *A. linearis* as well as to compare the trapping capacity of green and red rooibos tea. In addition, the antiglycation properties of fermented and unfermented rooibos hydroethanolic extracts were investigated. Selected individual compounds present in *A. linearis* and their potential intestinal metabolite phloroglucinol were also investigated in in vitro models with MGO and GO as the glycation agents.

## 2. Results and Discussion

The accumulation of AGEs is believed to be a factor contributing to the development of chronic metabolic diseases, such as metabolic syndrome, diabetes, and the vascular complications of diabetes [32]. Since in developed countries the prevalence of diseases associated with AGEs is increasing, research on the inhibitors of advanced glycation end-product formation has received much interest in recent years [33,34,35]. Natural products in particular have received a lot of attention due to their high level of safety and affordability.

Antiglycation activity has so far been reported for many plant raw materials, such as green tea [36,37], ginger [38], marjoram [39], Japanese star anise [40], buckwheat [41], and mung bean [42]. It has been shown that for some plant extracts, such as extracts of *Scutellaria alpina* L. and *Solidago altissima* L., antiglycation activity correlates with the content of flavonoids, and it is flavonoids that are largely responsible, through various mechanisms, for the ability to inhibit the formation of AGEs [43]. Individual compounds for which antiglycation and *α-dicarbonyl* trapping activity has been demonstrated include quercetin [44], kaempferol [45], rutin [46], hyperoside [47], myricetin [11], phlorizin [31], and many others.

Considering the already known beneficial effects of rooibos tea on the cardiovascular system, its safety of use, and ubiquity, as well as its richness in flavonoids—compounds that may exhibit anti-α-dicarbonyls and anti-AGE activity—we chose this plant material to examine whether it could potentially be effective as an agent in preventing the formation of advanced glycation end products. For this purpose, in the first place, the qualitative composition of *A. linearis* extracts was determined and the flavonoid content of hydroethanolic extracts in green and red rooibos was quantified (GRE and RRE, respectively). Based on the results, compounds with different chemical structures—the C-glycosides of the dihydrochalcones, flavones, and flavanones—were selected and investigated in the antiglycation test (MGO and GO models) and the α-dicarbonyl trapping assay. This study also covered an investigation of the antiglycation and trapping activity of hydroethanolic extracts and infusions of green and red rooibos. The final step was to identify the adducts formed as a result of trapping and propose the potential structures formed, as well as to demonstrate how structural differences in flavonoid compounds affect trapping ability. Our previous research included the study of flavonoid *O*-glycosides (e.g., rutin, isoquercitrin, and hyperoside) in this regard, while in this work we focused on *C*-glycosidic compounds [47,48,49].

### 2.1. Phytochemical Profile of Rooibos Extracts

In an effort to understand potential differences in the biological activity of green (GR) and red rooibos (RR), both GR and RR were subjected to phytochemical composition analysis. For this purpose, their infusions and hydroethanolic extracts were prepared as described in Section 3.3 and analyzed using UHPLC–ESI–MS. Identified constituents along with their *m/z* in negative ion mode and MS/MS fragments are presented in Table 1. The flavonoid profile was identical in both types of extracts. Therefore, further studies were conducted only with hydroethanolic extracts—GRE and RRE.

The infusions and hydroethanolic extracts from the fermented (RR) and unfermented (GR) raw material were rich in the dihydrochalcone C-glycosides aspalathin and nothofagin (peaks 21 and 20). Acetylaspalathin (peak 23) was observed only in RR. They also contained two pairs each of diastereoisomers of eriodictyol glucosides 1–4: (S)-6-C-, (R)-6-C, (S)-8-C-, and (R)-6-C-glucoside of eriodictyol (peaks 6–9). These compounds are formed by the natural process of aspalathin oxidation during fermentation and storage, and they have a dark brown color [50]. They are responsible for the color change of rooibos leaves from green to red brown during rooibos tea production [55]. The most abundant group of flavonoids found in fermented A. linearis was flavones. In analyzed rooibos extracts, positional isomers of apigenin and luteolin C-glycosides were identified: vitexin/isovitexin (peaks 14, 16) and orientin/isoorientin (peaks 11, 12), as well as di-C-glycosides such as carlinoside (peak 10). Orientin and isoorientin are formed from aspalathin by the intermediate flavanones 8-C- and 6-C-glucosides of eriodictyol [56]. Similarly, vitexin and isovitexin are formed from nothofagin by the respective C-glucosides of naringenin. The aglycones of flavones, such as luteolin and chrysoeriol, and flavanones, mainly eriodictyol, were also identified in the unfermented plant material (peaks 24–26), whereas flavonol O-glycosides, such as bioquercetin (quercetin-3-O-robinobioside, peak 15), rutin, hyperoside, and isoquercitrin (peaks 17–19) were detected in higher amounts in fermented A. linearis. The main difference in the composition of both types of rooibos is the amount of aspalathin, which is significantly higher in the non-fermented plant material. In the fermented raw material, it undergoes oxidation processes and is converted to eriodictyol-C-glucosides. However, both types of rooibos tea are rich sources of flavonoids, whose beneficial effect on health is undeniable.

Only the presence of aspalathin, vitexin, isovitexin, rutin, isoquercitrin, hyperoside, eriodictyol, luteolin and chrysoeriol was confirmed by standards; the other compounds were tentatively identified based on the literature.

### 2.2. Quantification of Flavonoids

In the next step, we determined the content of the main identified flavonoids in GRE and RRE. The concentration of dihydrochalcones, flavanones, flavones, and flavonols and the sum of flavonoids (expressed as mg/100 mL of hydroethanolic extract or 1 g of raw plant material) were quantified by the HPLC–DAD method using the external standards. We also converted the obtained results into micromolar concentrations. Table 2 shows the results of the quantitative analysis of flavonoid content.

The hydroethanolic extract of green rooibos (GRE) contained mainly dihydrochalcones at 46 mg/100 mL (1019.4 μM/L), predominantly aspalathin at 41.2 mg/100 mL (911.3 µM/L), and nothofagin at 4.7 mg/100 mL (108.2 μM/L), as well as flavone derivatives at 13.3 mg/100 mL (301.9 µM/L), especially C-glucosides of luteolin. The hydroethanolic extract of red rooibos (RRE) was especially rich in flavone derivatives: isoorientin, orientin, vitexin, isovitexin, and aglycones at a total of 7.3 mg/100 mL (184 µM/L), and a similar concentration of dihydrochalcones at 2.9 mg/100 mL (65.2 µM/L) and flavanones at 2.5 mg/100 mL (55.3 μM/L). Flavonols (3-O-robinobioside, 3-O-β-galactoside, and 3-O-β-glucoside of quercetin) were minor components of GRE and RRE. The sums of the flavonoids found in the two raw materials were extremely different. While unfermented rooibos contained as much as 65.5 mg/100 mL (1443.1 µM/L) of flavonoids, fermented rooibos contained only 13.6 mg/100 mL (320.2 µM/L). Thus, GRE was approximately 4.5 times richer in flavonoids and 15.6 times richer in dihydrochalcones than RRE.

Since we only had standards for aspalathin, vitexin, isovitexin, hyperoside, isoquercitrin, apigenin, luteolin, and chrysoeriol, the remaining compounds were calculated semi-quantitatively using available standards as described in Table 2.

### 2.3. Inhibition of Glycation by Rooibos Flavonoids and Related Compounds

In the present study, in vitro models using bovine albumin as the target protein and methylglyoxal or glyoxal as the glycation agent were used to evaluate the anti-AGE activity of the rooibos hydroethanolic extracts (GRE and RRE), aspalathin (ASP), vitexin (VT), isovitexin (IVT), eriodictyol (ER), and compounds with a similar structure, such as dihydrochalcone phloretin (PLT, phloretin is an aglycone of nothofagin) and phloroglucinol (PLG), which reflects the arrangement of the flavonoid A ring. Aminoguanidine (AG) and metformin (MET) were used as positive controls. AG is the most potent known inhibitor of non-enzymatic protein glycation [57], and MET is the first-choice drug in diabetic patients with a biguanide core; its anti-AGE activity has also been demonstrated in previous studies [58,59].

**Figure 1:**
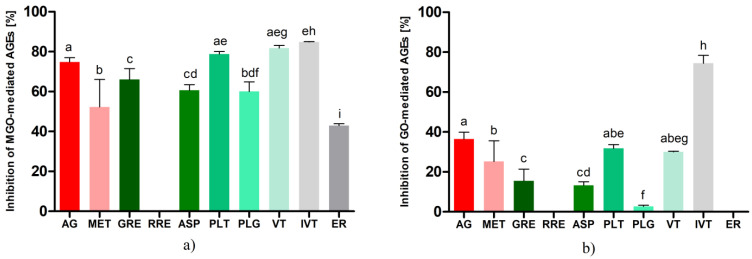
Antiglycation activity after seven days of incubation of bovine serum albumin with glycation agents (0.5 mM) and tested compound (1.5 mM) or hydroethanolic extracts (500 µL, DER 1:100) expressed as % inhibition of: (**a**) MGO mediated-AGE formation, (**b**) GO-mediated AGE formation. Results are representative of three experiments performed in triplicate ± SD. Values not sharing a common letter are significantly different at *p* < 0.05 according to Tukey’s multiple comparisons test. Abbreviations: AG, aminoguanidine; MET, metformin; GRE, green rooibos extract; RRE, red rooibos extract; ASP, aspalathin; PLT, phloretin; PLG, phloroglucinol; VT, vitexin; IVT, isovitexin; ER, eriodictyol.

As shown in Figure 1, the results obtained in the two glycation models differed markedly from each other. In the model using methylglyoxal, all the tested compounds showed rather high inhibitory activity against AGE formation. The greatest antiglycation activity was observed for apigenin-C-glucosides, i.e., isovitexin (84.92 ± 0.36%) and vitexin (81.77 ± 4.67%), and these values exceeded the activity of the aminoguanidine (74.81 ± 2.16%) used as a reference. Moreover, phloretin (78.76 ± 1.23%), being a dihydrochalcone structurally similar to aspalathin, exhibited potent anti-AGE activity at the aminoguanidine level. The main component of green rooibos, aspalathin, demonstrated slightly lower activity in inhibiting AGEs at 60.62 ± 2.86%, but this result was still above the activity of metformin (52.28 ± 13.82%). Phloroglucinol, whose structure forms the core of rooibos dihydrochalcones, exhibited antiglycation activity similar to aspalathin (60.03 ± 4.84%). This compound is used as an antispasmodic drug in some countries and showed the ability to capture reactive carbonyl species in earlier studies [60]. Eriodictyol was the least effective in inhibiting MGO-mediated AGEs (42.98 ± 2.65%).

Among the rooibos products, using identical sample volumes and drug–extract ratios (500 μL, DRE 1:400), only the hydroethanolic extract from unfermented A. linearis (GRE) demonstrated inhibitory activity against bovine albumin glycation in a model with MGO. The inhibitory activity of GRE was 66.06 ± 5.26%, which was comparable to that of aspalathin (difference not statistically significant).

In the glyoxal-induced glycation model, isovitexin was also the most potent antiglycation agent (81.94 ± 0.63%), and its inhibitory potential against AGE formation was more than two-fold higher than that of aminoguanidine (36.49 ± 3.42%) and three-fold higher than that of metformin (25.24 ± 10.37%). The differences were statistically significant. The glycation-inhibitory activity of phloretin and vitexin was similarly lower at 31.75 ± 1.94% and 30.06 ± 1.17%, respectively. Aspalathin revealed a glycation inhibitory effect of 13.32 ± 1.7%. The activity of phloroglucinol was marginal (2.72 ± 0.54%), and no inhibitory effect on glyoxal-triggered AGE formation was observed for eriodictyol.

In the model with GO as the glycation agent, as in the previous model with MGO, only GRE among the extracts tested exhibited an inhibitory effect on protein glycation. The inhibitory effect of the hydroethanolic extract of unfermented rooibos was observed at 15.58 ± 5.53%.

The beneficial effect of A. linearis in reducing glycation damage was reported by Kamakura et al. [61], who found that green rooibos extract effectively reduced AGE-induced reactive oxygen species levels. According to the work of Pringle et al. [62] an extract of unfermented A. linearis demonstrated the same glycation inhibition potential (about 50%) as aminoguanidine, the positive control in a glycation model with BSA as the target protein and glucose as the trigger for the glycation process. In our experiment, GRE activity was slightly but statistically significantly lower than that of aminoguanidine. Moreover, the fermented rooibos extract failed to display any antiglycation activity, whereas the same study by Pringle et al. [62] reported that fermented rooibos extract was superior to green rooibos extract in glycation inhibitory activity. Furthermore, in a study conducted by Kinae et al. [63], fermented red rooibos extract effectively inhibited the formation of glycated albumin by nearly 30%. In examining the antidiabetic potential of fermented A. linearis, these authors found a significant reduction in AGEs in the plasma of rats with streptozotocin-induced diabetes in response to alkaline and aqueous extract. Differences in the biological activity of fermented and unfermented rooibos, as well as their extracts, can be attributed to different extraction techniques and the content of individual active ingredients in the raw plant material depending on the place of harvest and the storage conditions.

According to our qualitative analysis of extracts, unfermented rooibos contained about 4.5 times more flavonoids than fermented rooibos. The most significant difference in composition was the high concentration of dihydrochalcones in the unfermented raw material (about 15.6 times higher than in the fermented one). This leads to the conclusion that the antiglycation activity of rooibos tea may be mainly attributed to the presence of dihydrochalcones, more precisely to the predominant component aspalathin. This conclusion seems to be supported by the fact that in both glycation models with MGO and GO used in the experiment, the activity of the green rooibos extract corresponded to the antiglycation activity of aspalathin (for MGO it was 66% vs. 60% and for GO it was 15% vs. 13%).

The individual compound that showed the strongest ability to inhibit AGEs in both models we tested was isovitexin. Kim et al. [64] reported the relatively high activity of isovitexin toward the inhibition of non-enzymatic protein glycation (IC50 = 85.2 μM, while IC50 for aminoguanidine used as a reference compound was 961 μM), additionally revealing the strong inhibitory activity of this compound against aldose reductase, which may be advantageous in the context of the prevention of diseases associated with high concentrations of AGEs. In our experiment, vitexin, particularly in the model with MGO as a glycation agent, exhibited high activity. These results are comparable to data from glycation models using MGO and glucose as inducers of protein glycation modifications. Peng et al. [42] reported more than 85% efficacy of vitexin in inhibiting AGEs in both in vitro models mentioned above. Drygalski et al. [65] reported recently on the antiglycation properties of phloroglucinol and found that it inhibited MGO- and GO-induced protein glycation by approximately 54% in both systems. Our results partially confirm these data in the methylglyoxal model, but such high activity was not observed in the used glyoxal model. These differences may be due to variations in the used concentrations and incubation conditions. The results for eriodictyol were consistent with those published by Liu et al. [66], who observed 40% inhibitory activity against methylglyoxal-induced AGEs. Under the conditions of our experiment, eriodictyol did not show inhibition against GO-mediated AGEs; data in the literature do not provide information on the activity of eriodictyol in this particular model. The percentage differences in the activity of each compound between the MGO-BSA and GO-BSA models were notable. This could be associated with the fact that during the process of the non-enzymatic glycation of proteins different types of AGEs are synthesized; some of them have fluorescence (e.g., PEN—pentosidine) while others do not show fluorescence properties (e.g., CML—carboxymethyl-lysine) [67]. The amounts and proportions in which they are produced depend on the glycation agent and the time of reaction. In a study by Nevin et al. [68], it was demonstrated that a model using human sperm as the matrix for glycation and GO as the inducer resulted in a statistically significantly higher amount of CML without fluorescent properties than when MGO was used as the trigger of glycation. In our experiment, we measured fluorescence. Therefore, it is likely that the activity results were lower in the model with glyoxal because some of the AGEs formed could not be included in the measurement. This should be considered a limiting factor of our study.

### 2.4. α-Dicarbonyl Compound Trapping and Adduct Analysis

The green and red rooibos hydroethanolic extracts (GRE and RRE), as well as selected individual flavonoids identified in the extracts and related compounds, were evaluated for α-dicarbonyl uptake capacity. After 1 h of incubation, the formation of adducts was investigated using the UHPLC–ESI–MS method. The Extract Ion Chromatogram (EIC) mode was used to search for pseudomolecular ions enlarged by the mass of one or two molecules of methylglyoxal (72 or 144 Da) and, similarly, glyoxal (58 and 116 Da). In this study, phloretin and phloroglucinol were used as reference compounds with recognized activity for the direct trapping of reactive α-dicarbonyls [31,69] A summary of the results including retention times, *m/z*, and the identification of adducts is shown in Table 3 for the reaction with methylglyoxal, and in Table 4 for the reaction with glyoxal. The source indicates whether adducts were observed in the reaction with extract or standard.

First, the ability to trap methylglyoxal and glyoxal was tested on reference compounds (phloretin, phloroglucinol) and individual flavonoids identified in the rooibos extracts (aspalathin, vitexin, isovitexin, and eriodictyol). The same tests were performed with GRE and RRE.

**Figure 2:**
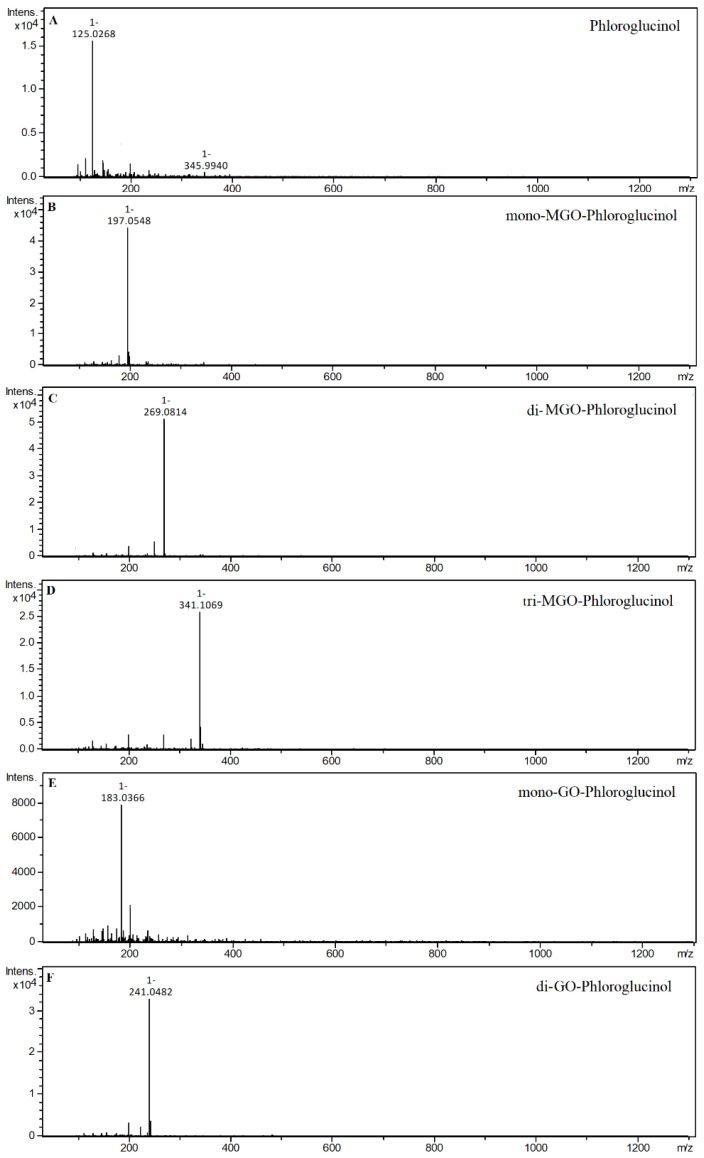
Mass spectra of phloroglucinol and its methylglyoxal/glyoxal adducts after 1 h of incubation in pH 7.4 phosphate buffer solution at 37 °C; (**A**), phloroglucinol; (**B**), mono-MGO-phloroglucinol; (**C**), di-MGO-phloroglucinol; (**D**), tri-MGO-phloroglucinol; (**E**), mono-GO-phloroglucinol; (**F**), di-GO-phloroglucinol. Other isomers are also possible.

**Figure 3:**
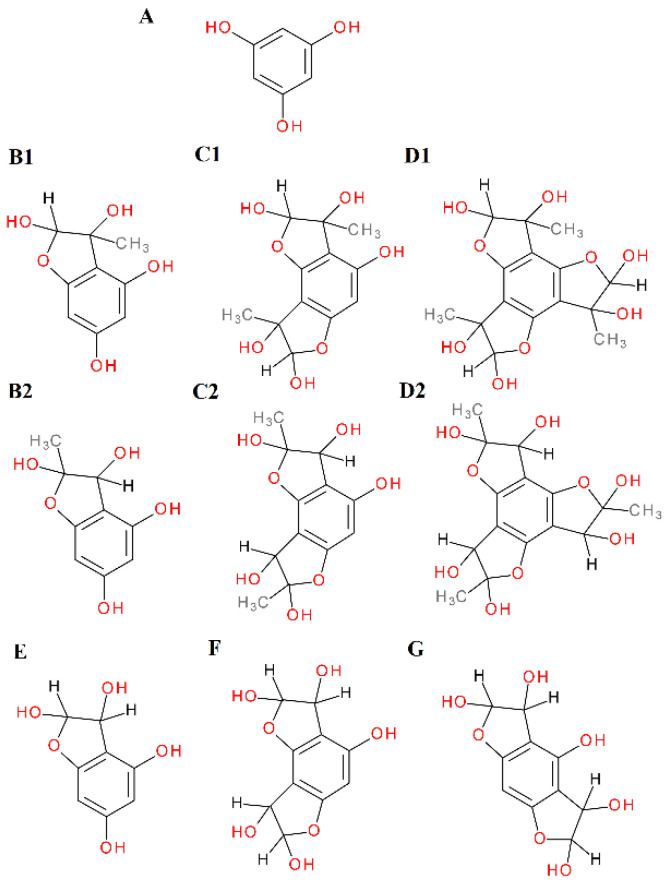
Proposals for chemical structures of adducts formed in the reaction of phloroglucinol with methylglyoxal/glyoxal after 1 h of incubation in pH 7.4 phosphate buffer solution at 37 °C; (**A**), phloroglucinol; (**B1**), hemiacetal form of mono-MGO-phloroglucinol; (**B2**), hemiketal form of mono-MGO-phloroglucinol; (**C1**), hemiacetal form of di-MGO-phloroglucinol; (**C2**), hemiketal form of di-MGO-phloroglucinol; (**D1**), hemiacetal form of tri-MGO-phloroglucinol; (**D2**), hemiketal form of tri-MGO-phloroglucinol; (**E**), mono-GO-phloroglucinol; (**F**), di-GO-phloroglucinol isomer a; (**G**), di-GO-phloroglucinol isomer b. Other isomers are also possible.

In the 1 h reaction of phloroglucinol with methylglyoxal, the formation of as many as seven new peaks on the chromatogram was observed. Two of these peaks with *m/z* 197 were identified as mono-MGO-phloroglucinol. This was confirmed by the presence of daughter ions with *m/z* 125 corresponding to phloroglucinol [M–72–H]^−^. Another three chromatographic peaks at *m/z* 269 were recognized as isomers of di-MGO-phloroglucinol. Their daughter ions with *m/z* 179 suggested the loss of one molecule of methylglyoxal and one molecule of water [M–72–18–H]^−^, while the ions with *m/z* 125 corresponded to the precursor ion, indicating the dissociation of two MGO molecules [M–144–H]^−^. The remaining two chromatographic peaks were annotated as tri-MGO-phloroglucinol isomers because the *m/z* of ions matched that of phloroglucinol increased by three methylglyoxal molecules and was 341. A similar decomposition pattern to that of di-MGO-phloroglucinol was observed herein, as ions at *m/z* 251 corresponded to tri-MGO-phloroglucinol with a loss of methylglyoxal and water [M–72–18–H]^–^. In addition, a characteristic ion at *m/z* 125 was also evident, representing the deprotonated phloroglucinol ion and implying the detachment of three methylglyoxal molecules [M–216–H]^–^. In the reaction of phloroglucinol with glyoxal, only two additional peaks appeared on the chromatogram after one hour. The first one was assigned as di-GO-phloroglucinol since its pseudo-molecular ion was at *m/z* 241, which is equivalent to the phloroglucinol precursor increased by 116 Da, meaning two GO molecules. Meanwhile, the second peak was designated as mono-GO-phloroglucinol since its *m/z* of 183 analogously corresponded to the attachment by phloroglucinol of one glyoxal molecule (58 Da). Mass spectra of the adducts formed by phloroglucinol and MGO/GO are shown in Figure 2 and the proposed chemical structures are shown in Figure 3.

The phloroglucinol structure determines the trapping reaction of α-dicarbonyls—the arrangement of -OH groups in the benzene ring in the meta position relative to each other provides the conditions for MGO/GO addition. The arrangement of phloroglucinol present in the structure of dihydrochalcones and other flavonoids—the A-ring with unsubstituted -OH groups (2 or 3) and unsubstituted carbons between them—is essential for the trapping activity of compounds. This conclusion is in line with the findings of a work by Liu and Gu [70]. They described the trapping ability of phloroglucinol derivatives as a metabolite of algae from the species *Fucus vesiculosus* L., obtaining results coinciding with our results. The authors of another publication describing the structural requirements for flavonoids to trap methylglyoxal stated that the A-ring is essential for MGO uptake, and the hydroxyl group at C-5 of the A-ring is strongly supportive of the methylglyoxal trapping capacity [71]. In their earlier work, the same authors indicated that the two unsubstituted carbons in ring A at positions 2′ and 4′ were the primary active sites for chalcones to trap MGO [31].

The trapping activity of MGO and GO by phloroglucinol differed. Phloroglucinol inhibited MGO-triggered glycation at the level of aspalathin and GRE and was more effective in this regard than was metformin. However, the compound’s ability to inhibit the formation of glycation end products with GO was negligible—only a few percent. The literature reports that phloroglucinol also exhibits antioxidant properties in the DPPH assay favorable for antiglycation activity [70], although there are also reports that phloroglucinol did not reduce H_2_O_2_-induced intracellular protein oxidation or carbonylation [72].

**Figure 4:**
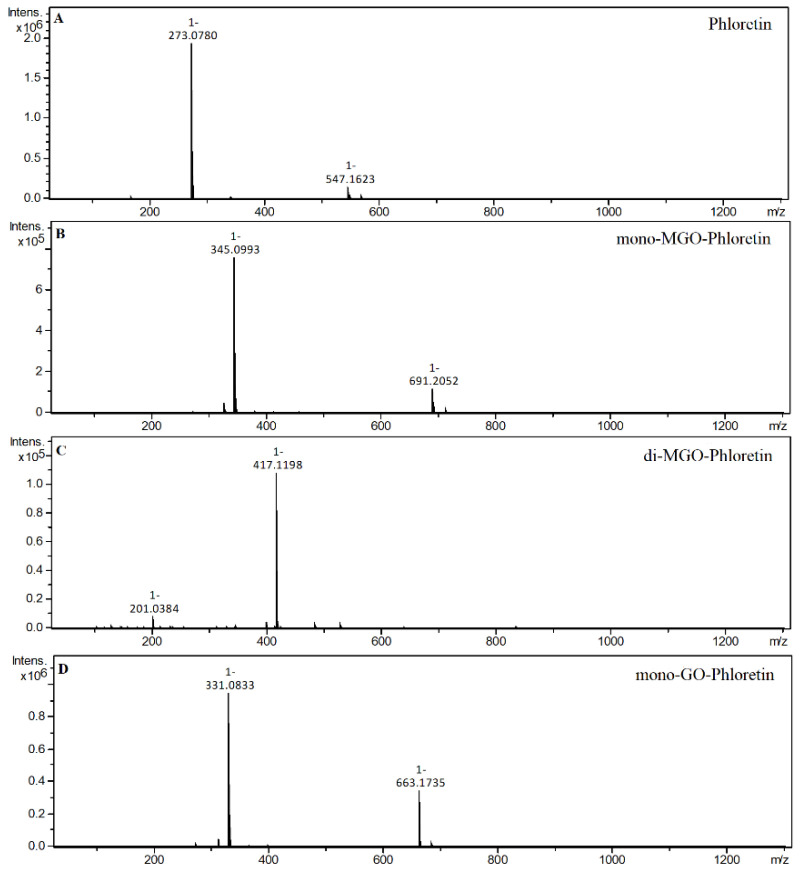
Mass spectra of phloretin and its methylglyoxal/glyoxal adducts after 1 h of incubation in pH 7.4 phosphate buffer solution at 37 °C; (**A**), phloretin; (**B**), mono-MGO-phloretin; (**C**), di-MGO-phloretin; (**D**), mono-GO-phloretin. Other isomers are also possible.

Dihydrochalcone with known trapping properties served as a reference compound in the experiment; phloretin produced three new chromatographic peaks after 1 h of incubation with methylglyoxal. Two of these peaks, whose pseudo-molecular masses were equal to *m/z* 417, were identified as di-MGO-phloretin, as their [M–H]^−^ corresponded to the phloretin deprotonated ion (*m/z* 273) increased by 144 Da (two MGO). The remaining formed adduct had an ion *m/z* of 345 and was designated as mono-MGO-phloretin since its mass corresponded to that of phloretin after the attachment of one MGO molecule. In the one-hour reaction of phloretin with glyoxal, the formation of a single monoadduct was observed with *m/z* equal to 331. The ion of the phloretin *m/z* 273 corresponded to the mass of the mono-GO-phloretin adduct minus the mass of one molecule of glyoxal [M–58–H]^−^. This is consistent with previous reports of phloretin’s ability to trap MGO and GO and confirms that the experimental conditions provided the potential for adduct formation. Mass spectra of the adducts formed by phloretin and MGO/GO are shown in Figure 4.

**Figure 5:**
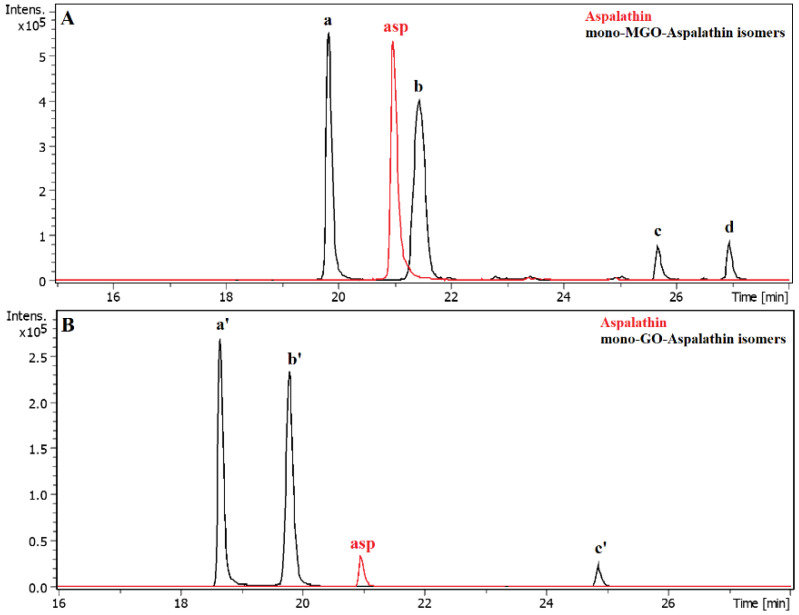
Representative LC chromatograms of aspalathin and its methylglyoxal (**A**)/glyoxal (**B**) adducts after 1 h of incubation in pH 7.4 phosphate buffer solution at 37 °C; (**A**), mono-MGO-aspalathin; (**B**) mono-GO-aspalathin; a, mono-MGO-aspalathin a; b, mono-MGO-aspalathin b; c, mono-MGO-aspalathin c; d, mono-MGO-aspalathin d; a’, mono-GO-aspalathin a; b’, mono-GO-aspalathin b; c’, mono-GO-aspalathin c; asp, aspalathin.

**Figure 6:**
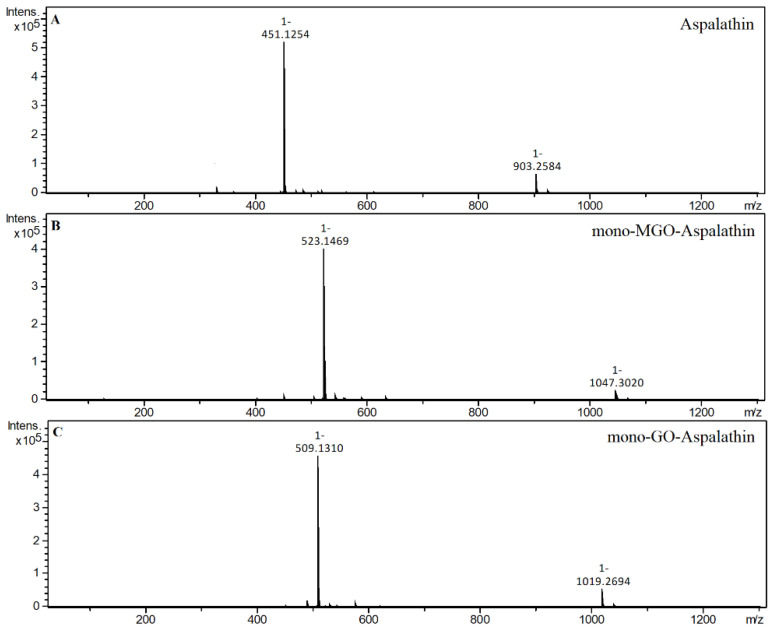
Mass spectra of aspalathin and its methylglyoxal/glyoxal adducts after 1 h of incubation in pH 7.4 phosphate buffer solution at 37 °C; (**A**), aspalathin; (**B**), mono-MGO-aspalathin; (**C**), mono-GO-aspalathin.

In the reaction of aspalathin with methylglyoxal, the formation of two new peaks on the chromatogram was observed after 1 h of reaction. Both were identified as mono-MGO-aspalathin due to an *m/z* ion mass of 523 corresponding to that of the aspalathin ion plus one molecule of methylglyoxal. In addition, the degradation profile corresponded to that observed for aspalathin in the extract, with the presence of daughter ions at *m/z* 505 [M–18–H]^–^ and 415 [M–36–72–H]^–^ reflecting the loss of one water molecule or two water molecules and one methylglyoxal molecule, respectively (described above). The presence of as many as four mono-MGO-aspalathin adducts was identified in GRE; two of them were identical to the aspalathin standard and the other two appeared on the chromatogram at around 26 and 27 min. These were probably isomeric forms, which were formed by different ways of attaching MGO to the phloroglucinol ring of aspalathin at the C-5′ position and the -OH group at the 4′ or 6′ position or the presence of an aspalathin isomer in the extract (see Table 1). In addition, the heterocyclic rings formed may have had a hemiketal or hemiacetal structure. In the reaction of aspalathin with glyoxal, after incubation, similarly to the reaction of GRE and glyoxal the formation of three chromatographic peaks with a pseudo-molecular ion at *m/z* 509 was observed, which corresponded to the attachment of one molecule of glyoxal and the formation of mono-GO-adducts [451 + 58–H]^−^. To the best of our knowledge, this is the first time that the trapping ability of methylglyoxal and glyoxal has been described for aspalathin. The example LC chromatograms of aspalathin and its MGO/GO adducts are shown in Figure 5. Mass spectra of the adducts formed by aspalathin and MGO/GO are shown in Figure 6 and the proposed chemical structures are shown in Figure 8.

The hydroethanolic extracts of green and red rooibos were also tested for their ability to trap MGO and GO. Only in the unfermented rooibos extracts was the ability of individual compounds to form adducts with methylglyoxal and glyoxal observed, and the fermented rooibos extracts did not demonstrate trapping properties.

After one hour of incubation of the GRE and methylglyoxal, the appearance of new peaks on the chromatogram was observed. Under the experimental conditions, monoadducts with MGO were formed by aspalathin and nothofagin. The reaction of methylglyoxal and aspalathin proceeded in the same way as for the standard described above, although after 1 h of incubation as many as four monoadducts of aspalathin and MGO were formed (see Table 3). The formation of adducts in the reaction with glyoxal, on the other hand, proceeded for aspalathin in exactly the same manner with both the extract and the single compound samples (Table 4).

**Figure 7:**
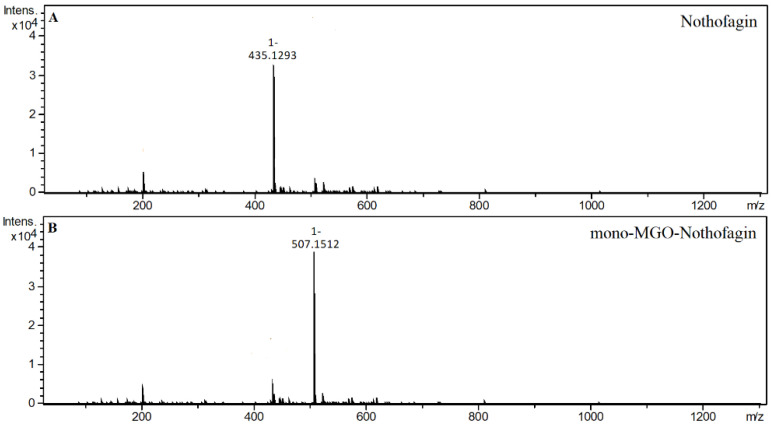
Mass spectra of nothofagin and its methylglyoxal adduct after 1 h of incubation in pH 7.4 phosphate buffer solution at 37 °C; (**A**), nothofagin; (**B**), mono-MGO-nothofagin.

**Figure 8:**
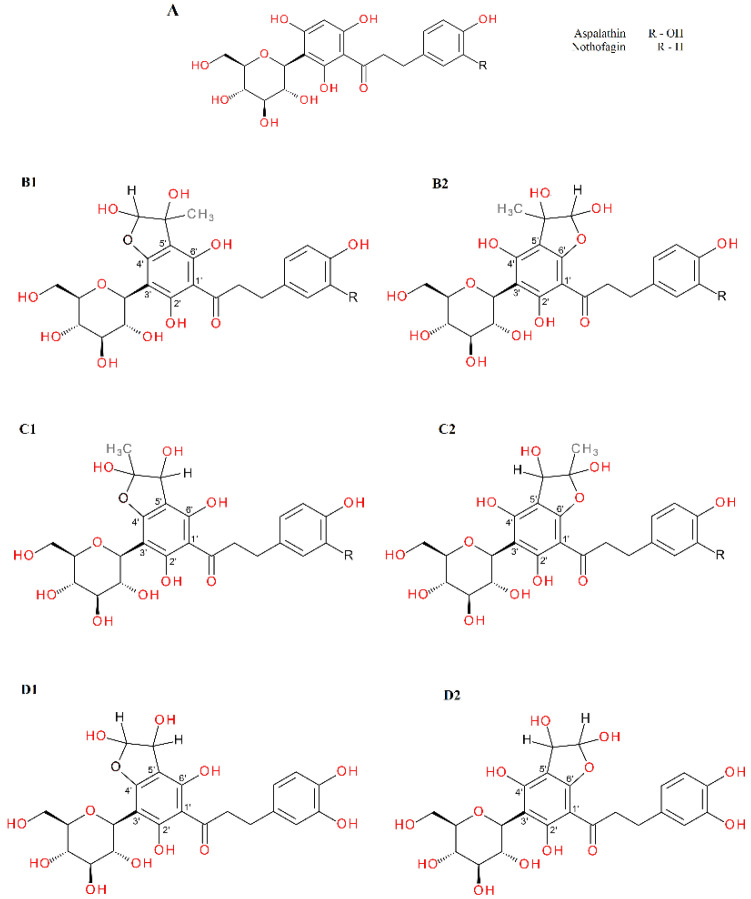
Proposals for chemical structures of adducts formed in the reaction of aspalathin/nothofagin with methylglyoxal/glyoxal after 1 h of incubation in pH 7.4 phosphate buffer solution at 37 °C; (**A**), aspalathin/nothofagin; (**B1**,**B2**), hemiacetal forms of mono-MGO-aspalathin/nothofagin; (**C1**,**C2**), hemiketal forms of mono-MGO-aspalathin/nothofagin; (**D1**,**D2**), mono-GO-aspalathin isomers. Other isomers are also possible.

Since we did not use a nothofagin standard, observations were made only for the nothofagin contained in the green rooibos extract. In the reaction with methylglyoxal, four new chromatographic peaks were formed, labeled as mono-MGO isomers of nothofagin. Their *m/z* was 507, which corresponded to the mass of the precursor ion *m/z* 436 increased by the mass of one molecule of methylglyoxal (72 Da). To the best of our knowledge, this is the first time that the trapping ability of methylglyoxal has been described for nothofagin. Mass spectra of the adducts formed by nothofagin and MGO are shown in Figure 7 and the proposed chemical structures are shown in Figure 8. In this experiment, nothofagin contained in the green rooibos extract was not observed to form adducts after 1 h of incubation with glyoxal.

**Figure 9:**
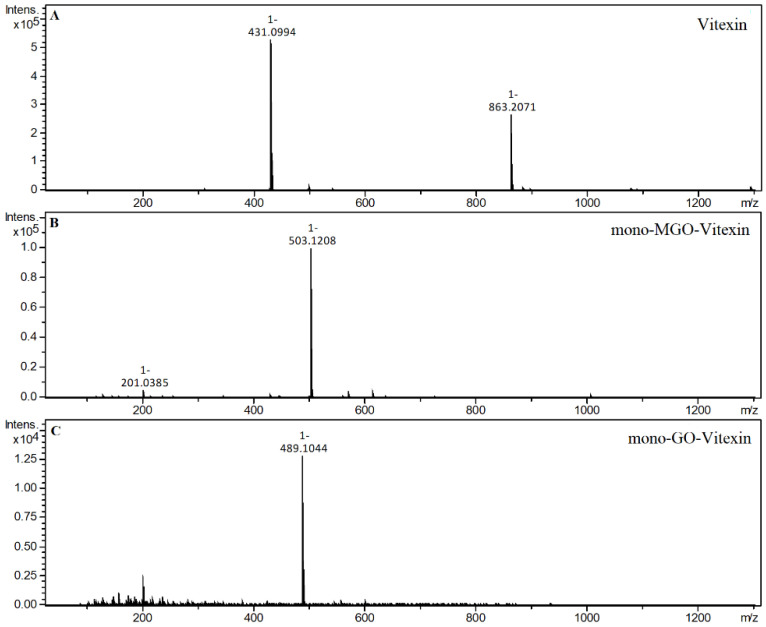
Mass spectra of vitexin and its methylglyoxal/glyoxal adducts after 1 h of incubation in pH 7.4 phosphate buffer solution at 37 °C; (**A**), vitexin; (**B**), mono-MGO-vitexin; (**C**), mono-GO-vitexin.

**Figure 10:**
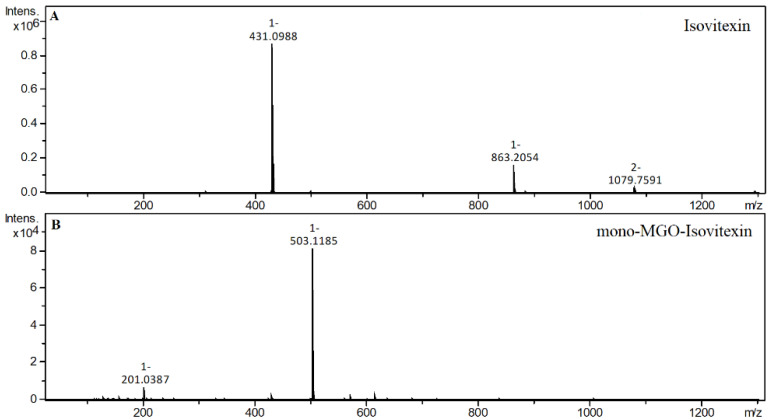
Mass spectra of isovitexin and its methylglyoxal adduct after 1 h of incubation in pH 7.4 phosphate buffer solution at 37 °C; (**A**), isovitexin; (**B**), mono-MGO-isovitexin.

**Figure 11:**
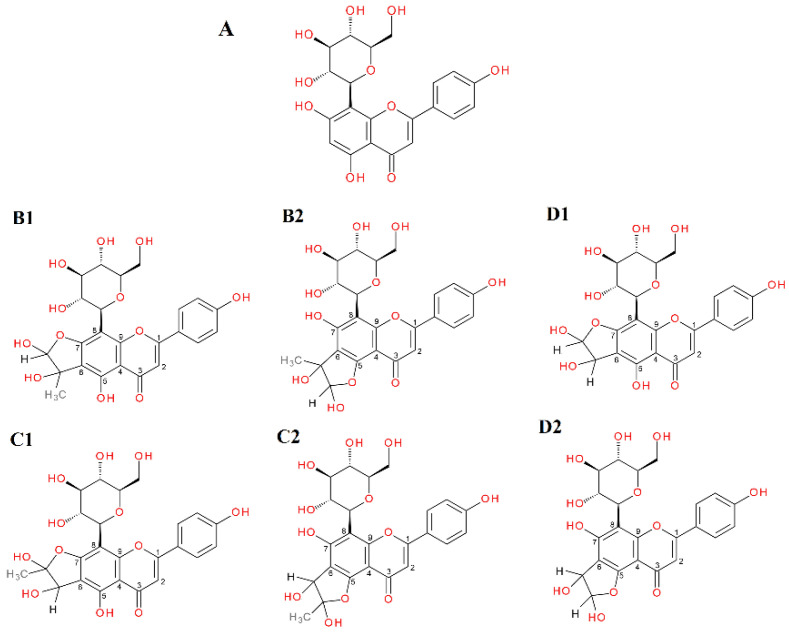
Proposals for chemical structures of adducts formed in the reaction of vitexin with methylglyoxal/glyoxal after 1 h of incubation in pH 7.4 phosphate buffer solution at 37 °C; (**A**), vitexin; (**B1**,**B2**), hemiacetal forms of mono-MGO-vitexin; (**C1**,**C2**), hemiketal forms of mono-MGO-vitexin; (**D1**,**D2**), mono-GO-vitexin isomers. Other isomers are also possible.

**Figure 12:**
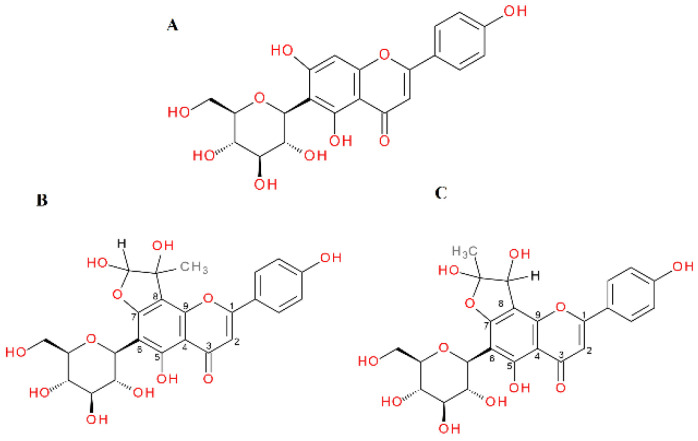
Proposals for chemical structures of adducts formed in the reaction of isovitexin with methylglyoxal after 1 h of incubation in pH 7.4 phosphate buffer solution at 37 °C; (**A**), isovitexin; (**B**), hemiacetal form of mono-MGO-isovitexin; (**C**) hemiketal form of mono-MGO-isovitexin. Other isomers are also possible.

The *C*-glycosylated flavones vitexin and isovitexin in a 1 h reaction with methylglyoxal produced two new chromatographic peaks, each with [M − H]^−^ at 503, suggesting that under the experimental conditions two mono-adducts each were formed for vitexin and isovitexin. These deprotonated ions corresponded to a vitexin and isovitexin precursor ion mass of 431 plus one molecule of methylglyoxal (72 Da). The decay spectrum revealed the presence of ions 413 and 311 formed by the loss of fragments with 90 Da and 120 Da, respectively, from the cross-ring cleavages of the hexose unit. This indicates that in the cleavage, the glucose moiety is degraded first rather than the heterocyclic structure of the methylglyoxal adduct. Mass spectra of the adducts formed by vitexin and isovitexin and MGO/GO are shown in Figure 9 and Figure 10, respectively. The proposed chemical structures of the adduct are shown in Figure 11 for vitexin and Figure 12 for isovitexin.

Previous studies on vitexin and isovitexin in MGO trapping reported inconsistent results. A study by Peng et al. [42] showed that vitexin and isovitexin lack the ability to direct methylglyoxal capture. However, another more recent study by Ni et al. [73] found that vitexin binds methylglyoxal to form adducts.

In the reaction of vitexin with glyoxal, two new peaks appeared on the chromatogram after one hour. Both were assigned as mono-GO-vitexin since their deprotonated ion at *m/z* 489 was equivalent to the mass of the vitexin precursor ion (*m/z* 431) increased by 58 Da, meaning one molecule of glyoxal.

In this experiment, isovitexin was not observed to form adducts after incubation with glyoxal. Both vitexin and isovitexin have one position each in the A-ring where the dicarbonyl MGO/GO can be attached; these are the C-6 or C-8 sites, respectively. If, as in the case of vitexin, there is a C-6 carbon, then two adducts can be formed with an -OH group at C-5 or C-7 with two forms of hemiketal or hemiacetal for each version. For isovitexin with an unsubstituted C-8 position, adducts are formed only with an -OH group at C-7 and again with two forms of hemiketal and hemiacetal. Therefore, isovitexin can have only two mono-adducts, while vitexin hypothetically can produce as many as four. Although it is most likely that only two are preferentially predominant, we do not actually know which. Isovitexin did not produce adducts with GO, so the C-8 position appears to be less favored, most likely due to differences in the C-8 surroundings and substituents at adjacent positions (-OH group free or in the C-ring heterocyclic system). As indicated, these reactions lead to the formation of at least several different stereoisomers of adducts of specific compounds with α-DCs, and some of them are preferentially formed due to their favorable electron configuration. At this stage of research, however, we are not able to determine their chemical structures specifically. For this purpose, further structural studies are necessary.

**Figure 13:**
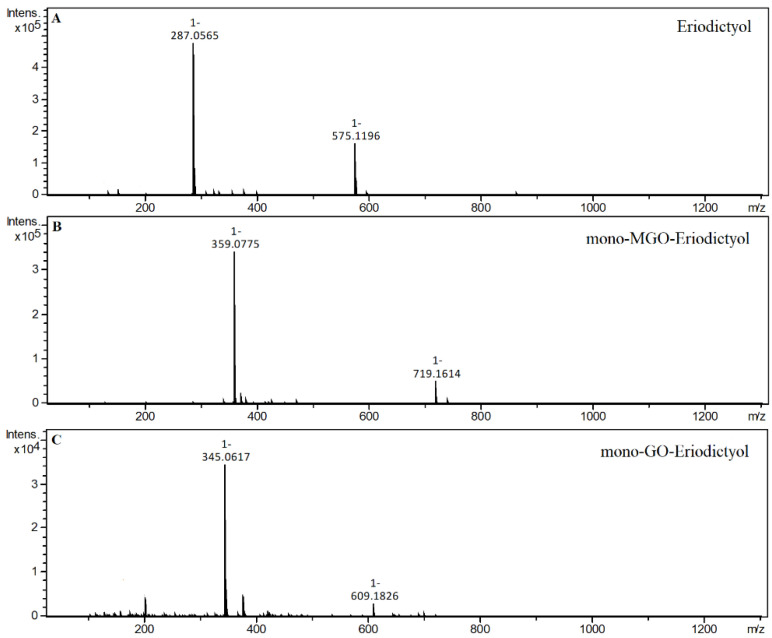
Mass spectra of eriodictyol and its methylglyoxal/glyoxal adducts after 1 h of incubation in pH 7.4 phosphate buffer solution at 37 °C; (**A**), eriodictyol; (**B**), mono-MGO-eriodictyol; (**C**), mono-GO-eriodictyol.

After the incubation of MGO with the only representative flavanones, i.e., eriodictyol, four additional chromatographic peaks were noted on the chromatogram labeled as mono-MGO-eriodictyol. Their pseudo-molecular ions were at *m/z* 359, which corresponds to the ion of the precursor *m/z* 287 enlarged by 72 Da (one molecule of MGO). For the reaction of eriodictyol with glyoxal, two additional peaks appeared on the chromatogram after 1 h of incubation. Both of these peaks were identified as mono-GO-eriodictyol (*m/z* 345, which corresponds to the mass of the eriodictyol ion increased by the mass of one glyoxal molecule). In *A. linearis*, C-glycosides of eriodictyol have been identified; according to the literature, glucose is substituted into the eriodictyol in a manner similar to that observed in the *C*-glycosides of apigenin and luteolin (at the C-6 or C-8 position of the A-ring). We did not have standards for specific eriodictyol-*C*-glycosides, so we used aglycone; however, based on the results for vitexin and isovitexin, it can be assumed that the eriodictyol-*C*-glycosides found in rooibos may also at least partially possess MGO- and GO-trapping properties. Further studies are needed for confirmation. Mass spectra of the adducts formed by eriodictyol and MGO/GO are shown in Figure 13.

Although three different subgroups of flavonoids—dihydrochalcones (aspalathin, nothofagin, and phloretin), flavones (vitexin and isovitexin) and flavanones (eriodictyol)—show different C-ring structures, all can effectively capture methylglyoxal, indicating that the C-ring of flavonoids may not play an important role in trapping MGO. By contrast, for nothofagin and isovitexin, we did not observe the ability to form adducts with glyoxal, although the two compounds have a different C-ring structure: Nothofagin as a dihydrochalcone has an open C-ring, while isovitexin as a flavone has a C-ring in closed form.

The structure of the B-ring does not seem to play a key role in the ability to trap α-dicarbonyls. In our test, both aspalathin with two -OH groups and phloretin and nothofagin possessing only one -OH group in the B-ring exhibited the ability to trap methylglyoxal. However, only phloretin was able to form diadducts with MGO, undoubtedly influenced by the fact that it does not have a substituted C-glucose in the A-ring. The importance of the A-ring structure of flavonoids was described above.

In summary, A. linearis extracts were rich in dihydrochalcones, flavanones, flavones, and flavonols. However, green rooibos extract had several times more flavonoids—especially aspalathin and nothofagin—than red rooibos extract. In the antiglycation assay, isovitexin demonstrated the highest activity in both glycation models with MGO and GO, and its activity was statistically significantly higher than that of aminoguanidine, a compound considered one of the most potent inhibitors of non-enzymatic glycation. Only green rooibos extract inhibited the glycation process at a level comparable to isolated aspalathin (no statistically significant difference), which suggests that it is aspalathin as the main component found in GR and GRE in the greatest amounts that determines the antiglycation activity. Indeed, red rooibos did not display the ability to inhibit protein glycation in any of the models. Some compounds tested, despite their ability to trap GO, showed no activity against glyoxal-induced glycation inhibition (e.g., eriodictyol). This was likely influenced by factors such as pH, reaction time, process temperature, and the fact that glyoxal is present in hydrated forms in solution (described in detail below). Others, despite their lack of GO-trapping ability, were able to inhibit the glyoxal-triggered glycation process, perhaps due to the strong antioxidant properties that are a component of the antiglycation effect. This study revealed that under the experimental conditions, all tested compounds demonstrated activity towards the direct trapping of methylglyoxal, while only aspalathin, vitexin, and eriodictyol exhibited the ability to trap glyoxal. Phloretin and phloroglucinol used as reference compounds showed trapping activity against both α-dicarbonyls. No MGO or GO adducts were observed for RRE, which is probably due to low concentrations of flavonoids in the extract, compared to GRE (320.2 μM/L vs. 1443.1 μM/L). In GRE, only dihydrochalcones were a source of MGO (aspalathin and nothofagin) and GO (aspalathin) adducts.

It is known that non-enzymatic antiglycation activity usually results from a number of different mechanisms of reaction. The literature reports that components of the inhibitory effect on the glycation process include antioxidant activity, the ability to chelate transition metals, and the capacity of compounds to trap α-DCs [39]. Frequently, these activities occur synergistically, especially in the group of natural compounds known as flavonoids. An example of a compound acting through all of these mechanisms is quercetin—well known in the context of inhibiting the glycation process, which is characterized by very strong antiradical activity, chelating properties, and the ability to trap methylglyoxal and glyoxal [44,74]. Given the above, it is important to keep in mind that often compounds exhibiting only some of these mechanisms may ultimately fail to exert a strong antiglycation effect, and sometimes compounds characterized by only one of these mechanisms may act as a potent antiglycation agent. This seems to be the case with isovitexin, which has proven most potent as an inhibitor of GO-induced glycation while lacking the ability to trap it. However, isovitexin has both the ability to chelate transition metals and strong antioxidant properties, which taken together can result in a strong antiglycation effect. Therefore, the scavenging of reactive dicarbonyls appeared to not be a major mechanism for isovitexin to inhibit protein glycation. However, given these considerations, another issue must be taken into account. Individual compounds reacted (or failed to react) differently with methylglyoxal and glyoxal. The discrepancy in the number of adducts formed in the reaction with MGO and GO is likely attributable to the fact that, as structural studies suggest, in aqueous solutions the main forms of GO are the hydrated monomer, dimer, and trimer. Consequently, the reactions of the investigated compounds with glyoxal were significantly slowed down by the transformation of the above-mentioned forms of GO to free GO [31]. Thus, there is a possibility that both isovitexin and nothofagin, which in our experiment did not demonstrate GO-trapping ability, may bind it under different experimental conditions. However, some other compounds from the group of flavones (vitexin) or dihydrochalcones (aspalathin and phloretin) have shown the potential to attach glyoxal molecules under given experimental conditions; therefore, it can be assumed that for isovitexin and nothofagin, this process takes place slightly less readily, perhaps due to the molecular spatial conditions. Further structural studies are needed to explain these discrepancies.

An important aspect that should be considered is the fact that aspalathin, despite its high antiglycation as well as trapping properties, has an extremely low level of bioavailability—at less than 1% [75]. However, aspalathin as a C-glycoside is metabolized with the involvement of bacterial enzymes in the large intestine, where low-molecular metabolites, such as phloroglucinol, have the chance to be absorbed into the circulation and can have a systemic antiglycation and trapping effect [76]. At this point, it should be mentioned that phloroglucinol is not an aspalathin-specific metabolite, but is a structural core of most flavonoids, which can exert their biological effects even with poor bioavailability of precursor flavonoids [77,78]. There is also evidence that the *C*-glycosides of flavonoids such as aspalathin and nothofagin present in *A. linearis*, which are metabolized in the large intestine, may exert local beneficial anti-AGE and anti-α-dicarbonyl effects in conditions such as irritable bowel syndrome. Bacterial metabolites that act deleteriously in excess on the digestive system and cause unpleasant symptoms in the form of irritation play a major role in these conditions [79]. One such bacterial metabolite is methylglyoxal. Thus, the use of dihydrochalcones and other flavonoids broken down in the intestine with the production of phloroglucinol can also exert a local effect by reducing the concentration of methylglyoxal as an irritant [80].

## 3. Materials and Methods

### 3.1. Plant Material

Dried, crushed leaves and apical parts of shoots of Aspalathus linearis (Burm. f.) R. Dahlgren plantations in South Africa were used to prepare infusions and hydroethanolic extracts. Two types of raw materials were used: unfermented (green rooibos, GR) and fermented (red rooibos, RR) purchased from the Polish tea manufacturer Oxalis (“OXALIS POLSKA SP. Z O.O.; Radzionków, Poland”).

### 3.2. Preparation of Extracts

Before the preparation of the extracts, both types of raw material were finely ground, each for 5 min, using an IKA A11B analytical mill (IKA Polska Sp. z o.o.; Warsaw, Poland). Then, 0.25 g of the powdered plant material was weighed on an analytical balance, placed in appropriately labeled volumetric flasks, and extracted with 25 mL of boiling water (infusions) or a mixture of water and ethanol (50%, *v/v*) for 15 min. The DER was 1:100. An ultrasonic bath (Bandelin Sonorex Digital 10P; Bandelin, Berlin, Germany) at 40 °C was used for hydroethanolic extraction. After 15 min, the extracts were centrifuged and then filtered using 0.22 μm diameter Durapore filters into vials. The filtrates were used for phytochemical analysis and in vitro tests. Hydroethanolic extracts from GR and RR were designated GRE and RRE, while infusions were designated GRI and RRI, respectively.

### 3.3. Chemicals

Methylglyoxal (MGO, 40% in water), glyoxal (GO, 40% in water), methanol (HPLC grade), acetonitrile (HPLC gradient grade and LC-MS grade), water (LC-MS grade), bovine serum albumin, DMSO, 98–100% formic acid, phloroglucinol (CAS No. 108-73-6), phloretin (CAS No. 60-82-2), eriodictyol (CAS No. 552-58-9), aspalathin (CAS No. 6027-43-6), aminoguanidine hydrochloride (CAS No. 16139-18-7), and metformin hydrochloride (CAS No. 1115-70-4) were purchased from Merck-Sigma-Aldrich (Sigma-Aldrich Sp. z o.o., Poznań, Poland). Vitexin (CAS No. 3681-93-4), isovitexin (CAS No. 38953-85-4), hyperoside (CAS No. 482-36-0), isoquercitrin (CAS No. 482-35-9), rutin (CAS No. 153-18-4), eriodictyol-7-O-β-glucoside (CAS No. 38965-51-4), apigenin (CAS No. 520-36-5), luteolin (CAS No. 491-70-3), and chrysoeriol (CAS No. 491-71-4) were purchased from Extrasynthese (Genay Cedex, France). NaCl, KCl, Na_2_HPO_4_, and KH_2_PO_4_ (reagent grade) were obtained from Chempur (Piekary Śląskie, Poland). Water used in the study was deionized. The stock solutions of standards for in vitro assays were prepared by dissolving the reference compound in 5 mL of a suitable solvent, filtered through hydrophilic Millex Syringe Filters Durapore 0.22 μm (Sigma-Aldrich, Poznań, Poland) and stored at −20 °C.

Stock solutions (1 mg/mL) for quantitative and semiquantitative analysis were made by dissolving 5 mg of flavonoid in 5 mL of methanol. Working standard solutions in the range of 10–250 g/mL (6 measurement points for each pattern) were prepared by mixing with 50% aq. methanol (*v/v*), filtered through hydrophilic Millex Syringe Filters, Durapore 0.22 μm (Sigma-Aldrich, Poznań, Poland) and stored at −20 °C.

### 3.4. Phytochemical Profile of Extracts

Phytochemical analysis of extracts was conducted using the Thermo Scientific Dionex UltiMate 3000 UHPLC system (Thermo Fisher Scientific; Waltham, MA, USA) incorporated with Compact ESI-QTOF-MS (Bruker Daltonics; Bremen, Germany), quaternary pump (LPG-3400D), and UltiMate 3000 RS autosampler (WPS-3000). Compounds were separated on a Kinetex C18 column (150 × 2.1 mm, particle size 2.6 μm) (Phenomenex; Torrance, CA, USA) and a temperature-controlled column compartment (TCC-3000) was used to maintain its temperature at 40 °C. Mobile phases consisted of 0.1% (*v/v*) formic acid in water (solvent A) and 0.1% (*v/v*) formic acid in acetonitrile (solvent B). The following gradient mobile phase program at a flow rate of 0.3 mL/min was used: 0–12 min, 97–65% A in B; 12–14 min, 65% A in B; 14–17 min, 65–20% A in B; 17–19 min, 20% A in B. Then, the system was returned to the initial settings and washed with 97% A in B until the system was stabilized before the next analysis. Negative ion mode (ESI−) was used for data acquisition. Nitrogen was used as a nebulizing gas at 210 °C temperature, 2.0 bar pressure, and 0.8 L/min flow rate. For internal calibration, sodium formate clusters (10 mM) were used. The injection volume was 2.5 μL. Additional operating conditions of the mass spectrometer were as follows: the capillary voltage was set at 5 kV, the collisional energy was 8.0 eV, and for the MS2 mode, it was 40 eV. The data processing was carried out using Compass Data Analysis software (Bruker Daltonics; Bremen, Germany). The same method was used to analyze the formation of MGO and GO adducts.

The content of aspalathin and other flavonoids in extracts was quantified using the HPLC–DAD method described in Section 3.4.

### 3.5. Quantification of Flavonoids

HPLC–DAD analysis was performed on a Smartline system (Knauer, Germany) with a pump (Managare 5000), dynamic mixing chamber (V7119-1), DAD 2800 detector, manual 6-port 2-channel injection valve (A1366), and column thermostat (Jetstream Plus). Data were processed using EuroChrom for Windows Basic Edition V3.05 (V7568-5). The separation was carried out on a Hypersil GOLD C18 column (250 × 4.6 mm, particle size 5 µm) with a C18 precolumn (10 × 4.6 mm, size 5 µm) (Thermo scientific, USA). The following eluents were used: C, 1.5% formic acid in water (*v/v*) and D, 1.5% formic acid in acetonitrile (*v/v*). HPLC gradient was as follows: 10%, 25%, 65%, 80% (D in C) at time points of 0-30-33-50 min. The flow rate was 0.9 mL/min, and the injection volume was 20 μL. The column was operated at 20 °C. The spectral measurements were made in the wavelength range 200–600 nm in steps of 2 nm. Dihydrochalcones and flavanones were analyzed at 280 nm, while flavones and flavonols were analyzed at 360 nm.

The HPLC–DAD method was validated according to ICH guidelines for linearity, detection and quantification limits, and intra- and inter-day precision. Calibration curves for the quantified flavonoids were determined from 6 measurement points, and double injections were performed for each concentration. The range of correlation coefficients of the calibration curves (r) used in the calculations was 0.999 to 0.9999.

Flavonoid content (mg/100 mL of hydroethanolic extract or 1 g of dry plant material) was determined using the external standard method from the areas of the corresponding peaks. Dihydrochalcones, flavanones, flavones, flavonols, and total flavonoids were calculated by summing the content of compounds from these (sub)groups. Nothofagin was calculated as an aspalathin equivalent. Similarly, orientin and isoorientin were expressed as vitexin and isovitexin, and eriodictyol-C-glucosides were expressed as eriodictyol-7-O-β-glucoside. The concentrations of other flavonoids were quantified using their corresponding standards. Mean content and standard deviations were calculated from five independent measurements. We also converted the obtained results into micromolar concentrations.

### 3.6. Glycation Process and Fluorescence Measurement of AGEs

#### 3.6.1. In Vitro Glycation Model

The glycation model was created based on a slightly modified published method [46]. In the proposed model, BSA serves as the protein and α-DCs serve as glycation agents. In short, 21.2 μM BSA was incubated with MGO or GO at 0.5 mM in 100 mM sodium phosphate buffer (pH 7.4) with 0.02% (*m/v*) sodium azide, which prevented microorganism growth in a test tube. Compounds investigated for antiglycation activity were added at a final concentration of 1.5 mM. Hydroethanolic extracts (DER 1:400) were added at a volume of 500 µL. Then, the reaction solution was incubated in simulated physiological conditions at 37 °C, shaken at 50 revolutions per minute for 7 days in closed vials secured with parafilm tape, and kept away from sunlight.

#### 3.6.2. Antiglycation Assay

The fluorescent intensity of α-DC-mediated AGEs formed during incubation was analyzed using a Synergy HTX Multi-Mode Microplate Reader (BioTek Instruments Inc., Winooski, VT, USA) at a wavelength of 360 nm for excitation (λ_ex_) and 460 nm for emission (λ_em_). Data processing was carried out using Gen5 Software (BioTek Instruments Inc., Winooski, VT, USA). The measurements from three experiments were all performed in triplicate, and the percent inhibition of AGE formation was calculated using the following equation:

Inhibition of α-DC-mediated AGEs [%] = –{1 − [(FI_1_)/(FI_0_)]} × 100

where FI_0_ is the mean fluorescence intensity of the blank sample and FI_1_ is the mean fluorescence intensity of the tested sample.

### 3.7. α-Dicarbonyl Trapping and Adduct Analysis

The direct MGO- and GO-trapping capacity of individual compounds and extracts was investigated according to the published method of Shao et al. [71] with slight modifications. Briefly, 0.6 mM of freshly prepared MGO or GO solution was incubated with 0.2 mM of an individual compound or 500 μL of *A. linearis* hydroethanolic extract (DER 1:400) and 0.1 M PBS (pH 7.4 was determined immediately before use) at 37 °C and shaken at 50 rpm for 1 h. The reaction was stopped by adding 2.5 μL of glacial acetic acid and transferring Eppendorf tubes with the collected samples to an ice water bath. Next, the samples were carefully filtered through hydrophilic Millex Syringe Filters (Durapore 0.22 μm; Millipore, Burlington, MA, USA) and analyzed using UHPLC–ESI–MS (described in Section 3.4) to test their capacity to form adducts with MGO and GO.

### 3.8. Statistical Analysis

All data are presented as mean ± standard deviation (SD). Data were analyzed using the Shapiro–Wilk test to assess the normality of distribution, followed by one-way analysis of variance (ANOVA) with Tukey’s multiple comparison test using the GraphPad Prism 9 software, and *p* values equal to or less than 0.05 were considered significant.

## 4. Conclusions

Dicarbonyls are ubiquitous in our bodies since they are metabolic intermediates and can be generated from glycolysis and lipid peroxidation. Reducing their concentration in the human body can be achieved through diet—primarily by eliminating excess simple sugars from the diet. However, since in some pathological conditions their overproduction is not only related to diet, attempts are also being made to reduce their concentration in the body through the use of MGO- and GO-trapping compounds, among others.

This study demonstrated that in addition to the many health properties attributed to green and red rooibos, this plant material may also be considered as a potential adjunctive anti-α-dicarbonyl agent preventing the onset and development of glycation-related conditions. Since there are currently no pharmacological strategies to decrease α-DC levels as an early preventive measure against AGE-induced diseases, it is important to search for adjunctive therapies. Our study indicates that green rooibos, probably due to its high concentration of aspalathin and nothofagin, exhibits MGO- and GO-trapping activity and antiglycation potential, unlike red rooibos. However, compounds present in both unfermented and fermented *A. linearis* can trap MGO and GO and inhibit the glycation process induced by α-DCs. Further studies using in vivo models are needed to confirm and expand our results.

## Figures and Tables

**Table 1:** UHPLC–ESI–MS data of red (RR) and green rooibos (GR) components in negative ion mode.

No.	Rt [min]	[M − H]^−^	MS/MS	Error [ppm]	Compound	Rooibos Type	Source
1.	8.47	255.0504	193, 165	−0.31	Pisidic acid	RR	[50]
2.	13.76	341.0879	179, 135	1.87	1-*O*-caffeoylglucose	RR	[50]
3.	14.75	325.0929	119	1.64	(*E)*-PPAG	RR	[51]
4.	15.08	289.0713	203, 109	0.29	Epicatechin	RR	[50]
5.	17.98	609.1450	-	−0.93	Luteolin-6,8-di-*C*-glucoside or galactoside	RR, GR	[52]
6.	18.12	449.1089	329, 193, 135	1.13	Eriodictyol-*C*-glucoside 1	RR, GR	[52]
7.	18.82	449.1092	329, 193, 135	1.80	Eriodictyol-*C*-glucoside 2	RR, GR	[52]
8.	18.89	449.1085	329, 193, 135	0.24	Eriodictyol-*C*-glucoside 3	RR, GR	[52]
9.	19.15	449.1074	329, 193, 135	−1.98	Eriodictyol-*C*-glucoside 4	RR, GR	[52]
10.	19.40	579.1329	399, 369	−3.62	Luteolin-*C*-glucoside-*C*-arabinoside (carlinoside)	GR	[53]
11.	20.44	447.0937	357, 327	2.14	Luteolin-*C*-glucoside 1 (isoorientin or orientin)	RR, GR	[52]
12.	20.62	447.0937	357, 327	2.14	Luteolin-*C*-glucoside 2 (isoorientin or orientin)	RR, GR	[52]
13.	20.96	451.1254	331, 209	3.01	Aspalathin	RR, GR	S
14.	21.80	431.0994	341, 311	3.65	Apigenin-*C*-glucoside 1 (vitexin)	RR, GR	S
15.	21.97	609.1461	300/301	0.87	Queretin-3-*O*-robinobioside (bioquercetin, bioquercitrin)	RR	[50]
16.	22.19	431.0988	341, 311	2.26	Apigenin-*C*-glucoside 2 (isovitexin)	RR, GR	S
17.	22.29	609.1461	300/301	0.87	Queretin-3-*O*-rutinoside (rutin)	RR	S
18.	22.41	463.0883	300/301, 271	1.39	Quercetin-3-*O*-β-galactoside (hyperoside)	RR	S
19.	22.70	463.0878	300/301, 271, 255	0.31	Quercetin-3-*O*-β-glucoside (isoquercitrin)	RR	S
20.	23.30	435.1293	345, 315	0.42	Nothofagin	RR, GR	[53]
21.	24.87	451.1254	331, 209	3.01	Aspalathin isomer	RR, GR	[52]
22.	25.34	461.1077	446, 298/299 283/284	−1.49	Methyl-luteolin-*O*-glucoside or galactoside (e.g., chrysoeriol-*O*-glucoside/ galactoside)	RR	[54]
23.	25.65	493.1343	361, 331, 209	−0.6	Acetylaspalathin	RR	[50]
24.	27.55	287.0565	151, 135	3.25	Eriodictyol	GR	S
25.	29.13	285.0405	285	2.05	Luteolin	GR	S
26.	32.82	299.0560	284	1.45	Chrysoeriol	GR	S

Rt, retention time; S reference standard; RR, red rooibos; GR, green rooibos.

**Table 2:** Flavonoid content in hydroethanolic extracts from green and red rooibos (GRE and RRE; 1:100, *m/v*) expressed in mg per 100 mL of hydroethanolic extract and 1 g of dry plant material and µM/L. The main components are highlighted.

Compound	GRE				RRE			
	mg/100 mL or 1 g		μM		mg/100 mL or 1 g		μM	
	Mean	SD	Mean	SD	Mean	SD	Mean	SD
DIHYDROCHALCONES:	45.95	1.28	1019.42	28.34	2.93	0.12	65.16	2.69
Aspalathin	41.23	1.28	911.26	28.25	2.36	0.13	52.25	2.78
Nothofagin ^1^	4.72	0.02	108.16	0.51	0.56	0.01	12.91	0.21
FLAVANONES:	1.86	0.22	41.39	4.93	2.49	0.10	55.27	2.29
Eriodictyol-6-*C*-glucoside ^2^	1.07	0.14	23.85	3.04	1.55	0.10	34.36	2.31
Eriodictyol-8-*C*-glucoside ^2^	0.79	0.17	17.54	3.85	0.94	0.03	20.91	0.57
FLAVONES:	13.26	0.14	301.87	3.31	7.30	0.35	184.04	4.53
Luteolin-6-*C*-glucoside (isoorientin) ^3^	4.93	0.03	109.96	0.63	3.14	0.13	70.04	2.89
Luteolin-8-*C*-glucoside (orientin) ^4^	4.09	0.07	91.30	1.64	2.35	0.08	52.52	1.72
Apigenin-8-*C*-glucoside (vitexin)	1.39	0.16	32.35	3.78	0.40	0.04	9.47	0.86
Apigenin-6-*C*-glucoside (isovitexin)	2.66	0.05	61.78	1.20	0.13	0.01	2.92	0.15
Luteolin	0.08	0.01	2.74	0.32	1.05	0.02	36.84	0.76
Apigenin + chrysoeriol ^5^	0.10	0.01	3.74	0.24	0.33	0.05	12.25	1.96
FLAVONOLS:	4.44	0.19	80.44	3.16	0.91	0.04	15.73	0.59
Queretin-3-*O*-robinobioside (bioquercetin, bioquercitrin) ^6^	2.98	0.20	48.95	3.23	0.82	0.03	13.44	0.50
Quercetin-3-*O*-β-galactoside (hyperoside)	0.72	0.04	15.23	0.76	0.06	0.01	1.36	0.23
Quercetin-3-*O*-β-glucoside (isoquercitrin)	0.75	0.02	16.25	0.43	0.04	0.00	0.94	0.04
SUM OF FLAVONOIDS	65.51	1.52	1443.13	33.64	13.63	0.53	320.20	7.92

^1^ calculated as aspalathin; ^2^ calculated as eriodictyol-7-*O*-β-glucoside; ^3^ calculated as isovitexin; ^4^ calculated as vitexin; ^5^ calculated as apigenin; ^6^ calculated as rutin; *n* = 5; green color, green rooibos; red color, red rooibos; the darker the color, the higher the content.

**Table 3:** Adducts of methylglyoxal and investigated compounds formed after 1 h of incubation in pH 7.4 phosphate buffer solution at 37 °C.

Compound	Source	R_t_ [min]	[M − H]^−^	Error [ppm]	MGO-Adduct/Precursor
Aspalathin	S	20.99	451.1254	3.01	Aspalathin
		19.85	523.1475	4.45	mono-MGO-aspalathin a
		21.45	523.1473	4.07	mono-MGO-aspalathin b
	GRE	20.99	451.1254	3.01	Aspalathin
		19.85	523.1475	4.45	mono-MGO-aspalathin a
		21.45	523.1473	4.07	mono-MGO-aspalathin b
		25.65	523.1469	3.31	mono-MGO-aspalathin c
		26.93	523.1449	−0.51	mono-MGO-aspalathin d
Nothofagin	GRE	23.33	435.1293	0.42	Nothofagin
		21.95	507.1519	3.24	mono-MGO-nothofagin a
		23.43	507.1512	1.86	mono-MGO-nothofagin b
		28.05	507.1521	3.63	mono-MGO-nothofagin c
Vitexin	S	21.81	431.0994	3.65	Vitexin
		20.55	503.1205	3.07	mono-MGO-vitexin a
		20.86	503.1208	3.66	mono-MGO-vitexin b
Isovitexin	S	22.19	431.0988	2.26	Isovitexin
		20.82	503.1191	0.29	mono-MGO-isovitexin a
		21.50	503.1185	−0.90	mono-MGO-isovitexin b
Eriodictyol	S	27.51	287.0565	3.25	Eriodictyol
		21.50	359.0772	1.40	mono-MGO-eriodictyol a
		21.57	359.0771	1.28	mono-MGO-eriodictyol b
		22.46	359.0777	2.80	mono-MGO-eriodictyol c
		24.67	359.0775	2.24	mono-MGO-eriodictyol d
Phloretin	S	31.59	273.0780	2.56	Phloretin
		27.05	417.1197	2.73	di-MGO-phloretin a
		27.46	417.1198	2.97	di-MGO-phloretin b
		28.82	345.0993	1.07	mono-MGO-phloretin
Phloroglucinol	S	2.02	125.0245	4.79	Phloroglucinol
		4.28	197.0549	2.02	mono-MGO-phloroglucinol a
		8.43	269.0813	−2.97	di-MGO-phloroglucinol a
		14.56	341.1067	−1.64	tri-MGO-phloroglucinol a
		14.82	269.0814	−2.60	di-MGO-phloroglucinol b
		15.01	341.1069	−1.05	tri-MGO-phloroglucinol b
		16.71	269.0812	−3.34	di-MGO-phloroglucinol c
		17.32	197.0540	−2.53	mono-MGO-phloroglucinol b

S, standard; GRE, green rooibos hydroethanolic extract; letters of the alphabet (a–d) represent different isomers of the same compound.

**Table 4:** Adducts of glyoxal and investigated compounds formed after 1 h of incubation in pH 7.4 phosphate buffer solution at 37 °C.

Compound	Source	R_t_ [min]	[M − H]^−^	Error [ppm]	GO-Adduct/Precursor
Aspalathin	S	20.99	451.1254	3.01	Aspalathin
		18.68	509.1311	3.10	mono-GO-aspalathin a
		19.81	509.1310	2.90	mono-GO-aspalathin b
		24.86	509.1316	4.08	mono-GO-aspalathin c
	GRE	20.99	451.1254	3.01	Aspalathin
		18.68	509.1314	3.69	mono-GO-aspalathin a
		19.81	509.1320	4.07	mono-GO-aspalathin b
		24.86	509.1318	4.47	mono-GO-aspalathin c
Nothofagin	GRE	23.33	435.1293	0.40	Nothofagin
Vitexin	S	21.81	431.0994	3.65	Vitexin
		19.03	489.1033	−0.01	mono-GO-vitexin a
		19.19	489.1044	2.23	mono-GO-vitexin b
Isovitexin	S	22.19	431.0988	2.26	Isovitexin
Eriodictyol	S	27.51	287.0565	3.25	Eriodictyol
		21.56	345.0813	0.73	mono-GO-eriodictyol a
		22.13	345.0810	−0.13	mono-GO-eriodictyol b
Phloretin	S	31.59	273.0780	2.56	Phloretin
		26.88	331.0833	4.59	mono-GO-phloretin
Phloroglucinol	S	2.02	125.0245	4.79	Phloroglucinol
		8.15	241.0482	2.9	di-GO-phloroglucinol a
		13.79	183.0366	1.36	mono-GO-phloroglucinol a

S, standard; GRE, green rooibos extract; letters of the alphabet (a–c)represent different isomers of the same compound.

## Data Availability

Data supporting reported results are available from the corresponding author.
